# HIV gp120 Induces, NF-κB Dependent, HIV Replication that Requires Procaspase 8

**DOI:** 10.1371/journal.pone.0004875

**Published:** 2009-03-16

**Authors:** Gary D. Bren, Sergey A. Trushin, Joe Whitman, Brett Shepard, Andrew D. Badley

**Affiliations:** 1 Division of Infectious Diseases, Mayo Clinic, Rochester, Minnesota, United States of America; 2 Program in Translational Immunovirology and Biodefense, Mayo Clinic, Rochester, Minnesota, United States of America; New York University School of Medicine, United States of America

## Abstract

**Background:**

HIV envelope glycoprotein gp120 causes cellular activation resulting in anergy, apoptosis, proinflammatory cytokine production, and through an unknown mechanism, enhanced HIV replication.

**Methodology/Principal Findings:**

We describe that the signals which promote apoptosis are also responsible for the enhanced HIV replication. Specifically, we demonstrate that the caspase 8 cleavage fragment Caspase8p43, activates p50/p65 Nuclear Factor κB (NF-κB), in a manner which is inhibited by dominant negative IκBα. This caspase 8 dependent NF-κB activation occurs following stimulation with gp120, TNF, or CD3/CD28 crosslinking, but these treatments do not activate NF-κB in cells deficient in caspase 8. The Casp8p43 cleavage fragment also transactivates the HIV LTR through NF-κB, and the absence of caspase 8 following HIV infection greatly inhibits HIV replication.

**Conclusion/Significance:**

Gp120 induced caspase 8 dependent NF-κB activation is a novel pathway of HIV replication which increases understanding of the biology of T-cell death, as well as having implications for understanding treatment and prevention of HIV infection.

## Introduction

The HIV env is a pleotrophic molecule which causes a range of effects on human cells, by ligating either the CD4 or chemokine receptors, env can cause activation, anergy, and/or apoptosis of the receptor bearing cell [1]. In addition, HIV env can independently enhance HIV replication [2], potentially through NFAT activation [3].

A prominent effect of gp120 on host cells is induction of apoptosis. Depending upon cell type and activation status, gp120 induced apoptosis can occur following CD4 crosslinking, or CXCR4 crosslinking, and despite early reports to the contrary, such apoptotic signaling cascades are caspase dependent [4]–[6]. The molecular signals which initiate gp120 induced apoptosis include the Fas/Fas ligand system and/or P38 MAPK [7]. In either situation, mitochondrial depolarization, release of cytochrome c, and formation of the apoptosome ensue [8]. This activates effector caspases 9 and 3, which function to activate initiator caspases such as caspase 8 to amplify the apoptotic cascade [9], and they also cleave host regulatory and structural proteins which promote the phenotypic characteristics of apoptosis.

Recently, a non-apoptotic role for procaspase 8 has become recognized: Nuclear factor κB (NF-κB) activation in response to antigen receptor, Fc receptor, or TLR2, 3, 4 ligation requires the presence of procaspase 8 [10], [11]. In response to these stimuli, procaspase 8 complexes with Iκκβ, resulting in phosphorylation and proteasomal degradation of Iκβα, followed by phosphorylation and nuclear translocation of p65 [10], [11]. More recently, TRAF6 has been suggested to bind caspase 8, promoting the movement of this complex into lipid rafts [12]. The interaction of TRAF6 with caspase 8 is enhanced by caspase 8 processing [12], suggesting that cleavage of the caspase 8 zymogen enhances the ability of caspase 8 to activate NF-κB. Also, the structurally related cFLIP can initiate NF-κB activation via TRAF2 [13], in a manner that is enhanced by its prior cleavage by caspase 8 [14].

Since HIV env initiates apoptosis and stimulates HIV replication, we questioned whether these events were related, and if so, whether procaspase 8 was involved in the enhanced HIV replication.

## Methods

### Cell Culture

Jurkat and I9.2 T cells (ATCC) as well as primary human CD4 T cells were grown in RPMI 1640 supplemented with 10% fetal bovine serum and 2 mM Glutamine. Primary human peripheral blood lymphocytes and cells from HIV-infected patients were obtained following informed consent. This protocol was reviewed and approved by the Mayo Clinic institutional review board (protocol #1039-03). 293T cells were cultured in DMEM plus 10% FBS and 2 mM Glutamine. HIV infections were performed using HIV IIIb using a high MOI of 2.5 mg/ml p24 in the infected supernatant. TNFα (R&D Systems, Minneapolis, MN) was used at 10 mg/ml where indicated. Anti-CD3 (Ortho Biotech, Rariton, NJ) and anti-CD28 (BD Pharmingen, San Jose, CA) were used at 1 ug/ml to mimic T cell receptor activation. Where indicated camptothecin (Sigma, St. Louis, MO) was used at 10 µm. Gp120 was purchased from Immunodiagnostics (Woburn, MA), SDF was purchased from R&D systems (Minneapolis, MN).

CD4 T cells were isolated (98% CD4 T cells as determined by flow cytometry) from the blood of healthy volunteer blood donors by using RossetteSep CD4 enrichment cocktail in accordance with the manufacture's protocol (StemCell Technologies, Vancouver, British Columbia, Canada). The expression of activation markers as CD69 and HLA-DR on resting CD4 T cells were determined by flow cytometry.

Resting CD4 T cells (CD4+/CD69−/HLA-DR-) were incubated with HIV-1 ×4 gp120IIIB (Immuno Diagnostics, Inc. Woburn, MA) or gp120 IIIB pretreated with soluble CD4 (1∶2 ratio) (Immuno Diagnostics, Inc. Woburn, MA) at concentrations of 1 µg/ml/2×10^6^ cells for 30 minutes on ice and then incubated for 24 hours at 37°C. The following day, cell death was analyzed by staining with AnnexinV-Cy-5 following the manufacturer's instructions (BD Biosciences). All experiments were performed at least three times.

### PCR, Plasmids and Transfection

Nef was PCR amplified from DNA extracted from normal or HIV IIIB infected Jurkats and I9.2 cells using primers and protocol described by Zhu, et al [15]. The procaspase 8 cDNA was a gift from Dr. Marcus Peter. The genes encoding p50, p65, IκBα, and IKKγ were PCR amplified from a human cDNA library and cloned into pcDNA3 (Invitrogen, Carlsbad, CA). The Casp8p43 construct was created by site directed mutagenesis of the serine at amino acid 375 to a stop codon. A dominant negative IκBα was created to block IKK activation of NF-κB by PCR generated mutagenesis of serines 32 and 36 to Alanine [16]. For mammalian expression, the constructs were cloned into either pEGFP or pcDNA3 (Invitrogen, Carlsbad, CA). All plasmids were confirmed by DNA sequence analysis and tested for expression prior to experimental use.

The luciferase reporter constructs HIV-1 LTR Luc and HIV-1 LTR ΔKB Luc have been previously described [17]. The TK-Renilla plasmid, purchased from Promega (Madison, WI), was used as an internal control in all reporter plasmid transfections. Transfection efficacies of 30–40% are routinely achieved in these experiments as assessed by parallel transfections with expression vectors encoding fluorescent proteins. Results are expressed as luciferase per Renilla expression in order to normalize for variability between transfection efficiency and cell viability, between experimental groups, and between experiments.

Jurkat and I9.2 T cells were transfected with 1 ug plasmid/10∧6 cells using an Electro Square Porator T820 (BTX, San Diego, CA) at 300 volts for 10 msec. Transfection of primary CD4 T cells was done using AMAXA. For the caspase 8 reconstitution experiments, I9.2 Jurkat cells were electroporated with mammalian expression vectors coding for GFP or GFP-caspase 8 along with the HIV LTR-Luciferase and TK-Renilla reporter plasmids and incubated 18 hours at 37°C. 10∧6 cells were then incubated with or without either 1 ug/ml HIV IIIB gp120 + 0.5 ug.ml soluble CD4 or 20 nM SDF-1α for 24 hours at 37°C. The cells were then harvested and HIV LTR activity was measured using the Dual Light Assay Kit (Promega, Madison, WI) as per the manufacturer's recommendations.

### Stable Cell Lines

The Jurkat T-derivative cell line, I9.2, deficient in procaspase 8 protein expression, was transfected with expression vectors encoding for either green fluorescent protein (GFP), or procaspase 8 wild type conjugated to GFP (GFP-casp 8 WT). After transfection, the cells were placed in media containing 800 ug/ml Geneticin and cultured for 14 days passing cells every three days with fresh media and Geneticin. The cells were then checked for GFP expression by fluorescent microscopy and for protein expression by western blotting. The transfected cells were maintained in media containing 500 ug/ml Geneticin. For electroporation experiments the Geneticin was removed 24 hours in advance.

### Nuclear Protein Extraction and Electromobility Shift Assay (EMSA)

293T cells were transfected with empty vector or plasmids coding for full length procaspase 8 or p43 caspase 8. After six hours at 37°C, nuclear proteins were harvested as previously described [18], 5 ug of nuclear protein was allowed to bind to a 32-P labeled, double stranded, oligonucleotide encompassing the NF-κB binding site of the HIV-1 LTR (5′-ACAAGGGACTTTCCGCTGGGGACTTTCCAGGG-3′) at room temperature in the presence or absence of antibodies specific for the NF-κB transcription factors p50 or p65 (Rel A) (Santa Cruz Biotechnology, Inc., Santa Cruz, CA). The DNA-nuclear protein complexes were run on a 6% non-denaturing polyacrylamide gel at a constant voltage of 170 volts. The gel was then dried onto filter paper and exposed to autoradiography film.

### HIV-1 p24 Antigen Assay

Culture supernatants were harvested and assayed with the RETROtek HIV-1 p24 ELISA kit (ZeptoMetrix Corp., Buffalo, NY) for the presence of the HIV-1 antigen p24.

### Statistical Analysis

Standard error was calculated by standard deviation divided by ‘n’. Comparison was made using T tests with significance being defined as p<0.05.

### Western Blot

Human CD4 T-cells (10∧6) were incubated with or without 1 ug/ml HIV IIIB gp120 + 0.5 ug/ml soluble CD4 (Immunodiagnotics Inc., Woburn, MA), or 0.1 ug/ml anti-CD95 (CH11 clone from Upstate Biotechnologies, Billerica, MA) at 37°C for 48 hours. The cells were harvested and lysed in 20 mM Tris/HCl pH 7.5, 150 mM NaCl, 0.1% Triton X-100 with protease inhibitors (2 ug/ml Aprotinin, 10 ug/ml Leupeptin, 2 ug/ml Pepstatin, and 1 mM PMSF) for ten minutes on ice. The lysates were centrifuged at 12,000×g and the supernatants run on 10% PAGE, transferred to PVDF membrane and western blotted with antibodies specific for human caspase 8 (c-14 clone form Dr. Marcus Peter) and B-actin.

## Results

### HIV gp120 potently induces HIV replication

**Figure 1:**
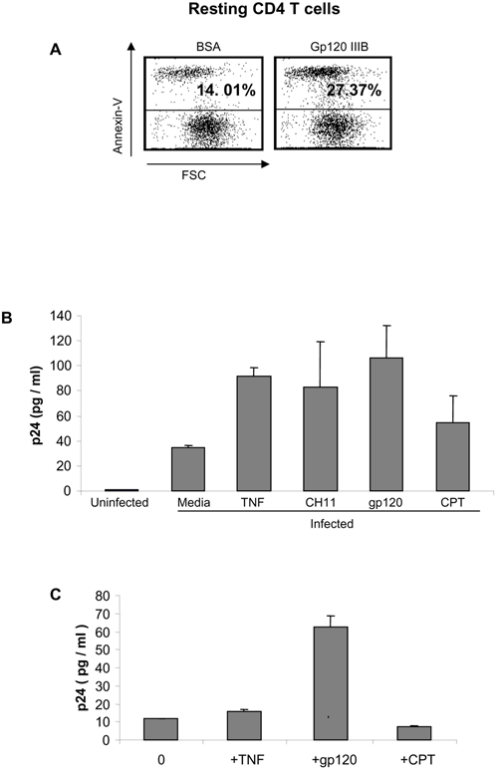
HIV gp120 induces HIV replication in resting CD4 T cells. (A) Primary CD4 T cells were harvested, sorted into the resting subset, and stimulated with gp120, and analyzed for death. (B) Primary CD4 T cells harvested from uninfected donors were infected or not with HIV IIIb and cultured for seven days. The infected cells were then divided and cultured at 10∧6/ml with media, 10 ng/ml TNF, 0.5 ug/ml CH11 anti-Fas receptor antibody, 1.0 ug/ml gp120 IIIb, or 10 uM camptothecin. A p24 ELISA was performed on culture supernatants as per the manufacturer's instructions. (C) PBLs were harvested from HIV-positive donors. The cells were washed three times and cultured over night in RPMI 1640 plus 10% FBS. The cells were then washed three additional times and cultured at 1×10∧6 without stimulation, or with 10 ng/ml TNF, 0.5 ug/ml CH11 anti-Fas antibody, 1.0 ug/ml HIV IIIb gp120, or 10 uM camptothecin for 18 hours at 37°C. Supernatants were tested for the presence of the HIV protein p24 by ELISA.

Gp120 has been proposed to mediate death of uninfected CD4 T cells; however whether gp120 kills resting CD4 T cells in the absence of activation is controversial. Therefore, we first analyzed the effect of gp120 on resting CD4 T cells from HIV negative donors (Figure 1A). Primary T cells from HIV-negative donors were first infected with HIV, and then stimulated with stimuli known to induce apoptosis: TNF, an agonistic anti-Fas antibody (CH11), viral gp120, and camptothecin (CPT). Doses were chosen that induced similar degrees of apoptosis (Figure 1B). Strikingly, each of these proapoptotic signals induced HIV replication. In addition, those stimuli that induce procaspase 8 activation (TNF, CH11, and gp120) induced more viral replication than does camptothecin, which acts to induce mitochondrial apoptosis independent of caspase 8 activation [19]. Equally striking is the observation that gp120 induced the greatest p24 production of all the caspase 8 dependent stimuli. We next assessed the impact of gp120 and other apoptosis inducers on HIV replication in primary CD4 T cells from HIV-infected patients (Figure 1C). CD4 T cells from four HIV-infected donors were isolated by negative selection, cultured overnight, and then stimulated with gp120 (IIIb), and other apoptosis inducing agents as indicated, and analyzed for p24 production 18 hours later (Figure 1C). No correlations were observed between *in vitro* p24 production, and CD4 count or viral load. HIV gp120 induced maximal HIV replication, yet in this context, HIV replication reflected virus produced from cells infected with HIV *in vivo*. Clearly, HIV gp120 is a potent inducer of HIV replication in primary HIV-infected CD4 T cells, although the mechanisms underlying this enhanced replication are undefined.

### HIV Replication that Occurs During Apoptosis is NF-κB Dependent

NF-κB is a transcription factor that is a key regulator of inflammation, apoptosis, immune activation, cell proliferation, and viral replication. Multiple stimuli are capable of activating NF-κB including, but not limited to, proinflammatory cytokines (e.g., TNF-α), activating cellular receptors (e.g., TCR), viral proteins (e.g., EBV-LMP-1), DNA cleavage, chemotherapeutics (doxorubicin), and oxidative stress. We have previously assessed the involvement of NF-κB family members in HIV replication that occurs during the peak of virus-induced death by demonstrating that a super repressor form of IKBα blocks HIV replication, thereby implicating NF-κB family members as the dominant transcriptional mechanism that drives such HIV replication [20]. We next asked whether gp120-induced HIV replication is mediated by transcriptional upregulation.

**Figure 2:**
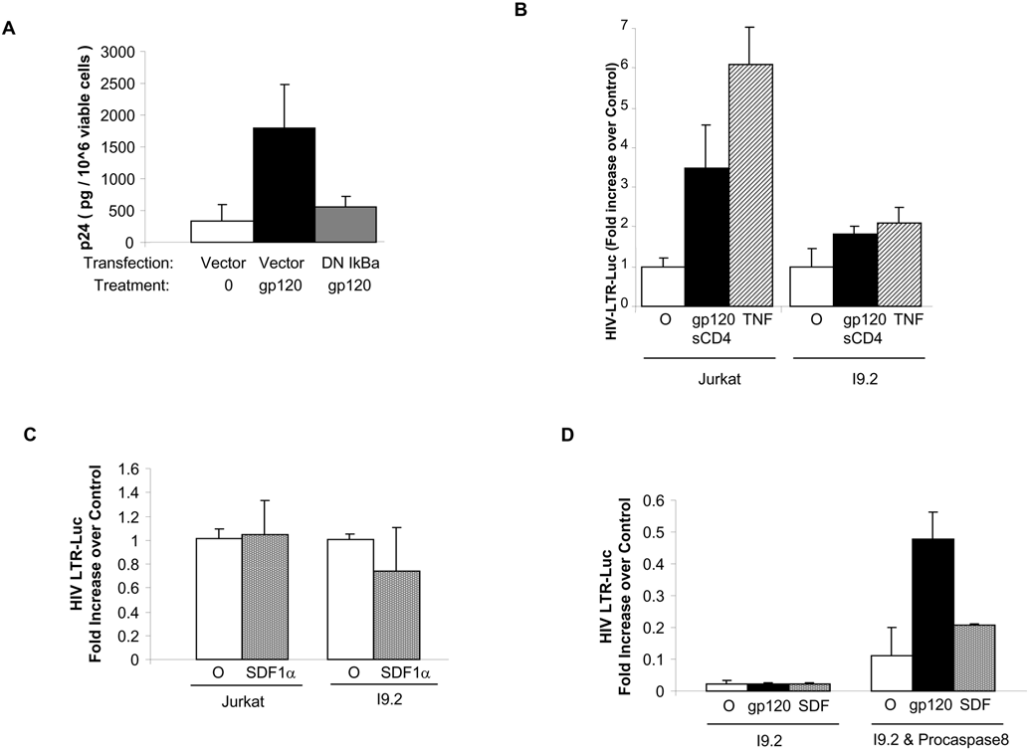
Enhanced HIV replication by gp120 requires NF-κB and caspase 8. (A) Primary CD4 T cells from HIV-infected patients were transfected with vector control or dominant negative IκB and treated with gp120 as indicated. P24 was measured the following day. (B) Jurkat or I9.2 cells were transfected with a luciferase reporter under the control of the HIV LTR promoter and a Renilla expressing plasmid as an internal control and cultured 18 hours with or without HIV gp120 (5 ug/ml) plus sCD4 (2.5 ug/ml) where indicated TNF was used as a positive control. The cells were harvested and the luciferase activity was measured and expressed in terms of fold increase over the control. (C) Jurkat or I9.2 cells transfected as above and treated with SDF as indicated and HIV LTR Luc measured, normalized to TK-Renilla, and expressed as fold increase over control. (D) I9.2 cells were transfected with empty vector (left) or procaspase 8 (right), stimulated with gp120 or SDF, and HIV LTR activity measured.

First, we treated cells from HIV-infected patients with gp120, in the presence or absence of a dominant negative form of the NF-κB inhibitor IκBα. Cells were then cultured overnight and p24 production monitored. Cells treated with gp120 and transfected with vector control had a significant induction of p24 compared to those not treated with gp120. The cells transfected with DN IκBα had significantly impaired p24 production, indicating that gp120 induction of HIV replication is NF-κB dependent (Figure 2A).

Next, we transfected Jurkat T cells with a luciferase reporter construct under control of HIV LTR. In parallel, TK-Renilla was co-transfected as a control for transfection efficiency. Cells were then stimulated with gp120/sCD4 in order to isolate the effect of gp120 signaling to signaling only through the CXCR4 receptor; and luciferase expression was measured and normalized to TK-Renilla (Figure 2B). Indeed, gp120 treatment increased luciferase expression demonstrating a transcriptional mechanism underlying the effects of HIV gp120 on HIV replication. This effect required caspase 8 because the Jurkat-derived caspase 8 deficient cell line, I9.2, which expresses CXCR4 (MCF in isotype Jurkat 31.8; CXCR4 in Jurkat  =  73.6; MCF isotype in I9.2  =  31.3 CXCR4 in I9.2  =  69.4,) failed to upregulate HIV LTR activity after treatment with gp120 (Figure 2B). Of interest, I9.2 cells also failed to upregulate LTR activity in response to TNF, which also induces caspase 8 activation. Finally, we observed that only gp120, but not SDF-1α, which is the natural ligand for CXCR4, caused upregulation of HIV LTR activity in a caspase 8 dependent manner (Figure 2C). The inability of caspase 8 deficient cells to respond to gp120 by increasing HIV LTR activation is specifically due to the lack of caspase 8 since I9.2 cells reconstituted with procaspase 8 have a restored response to gp120 stimulation (Figure 2D). We therefore focused attention on gp120 induced caspase 8 dependent HIV LTR activation.

### Casp8p43 Cleavage Intermediate Activates NF-κB Driven Activation of the HIV LTR

**Figure 3:**
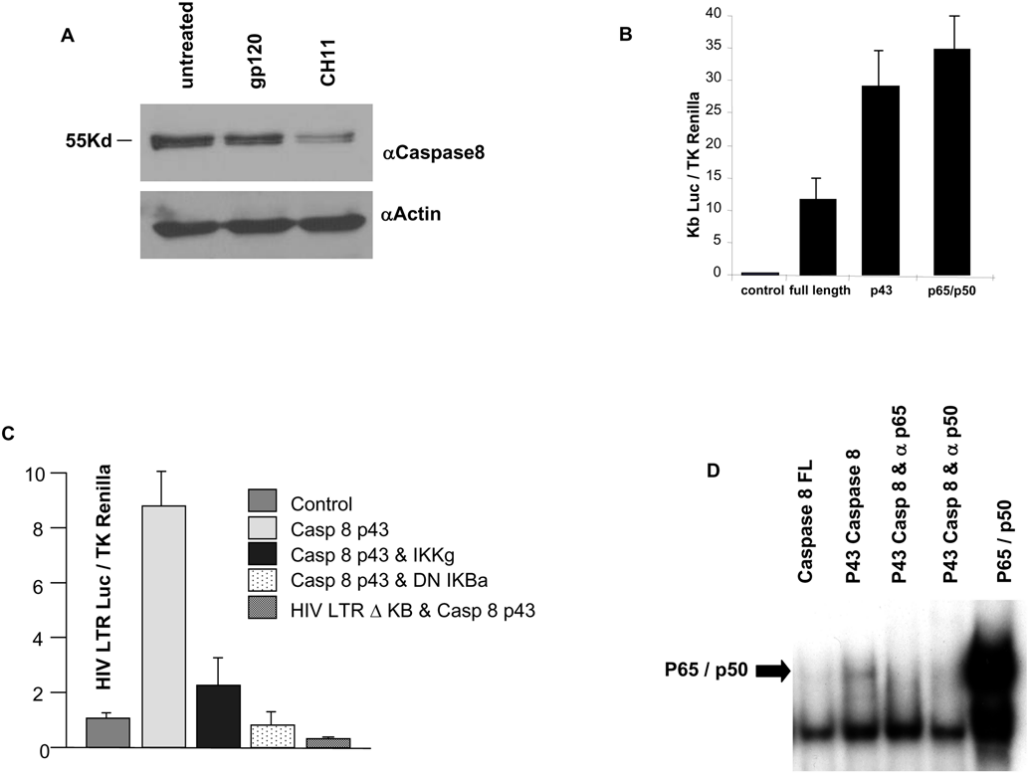
Casp8p43 induces NF-κB dependent HIV LTR transcription. (A) Jurkat T cells were treated with gp120 or CH11 anti-Fas antibody and analyzed for caspase 8 cleavage by western blot. (B) Jurkat T cells transfected with κB luciferase reporter plasmid, TK-Renilla as a control for transfection efficiency, and either control vector or vector expressing full length caspase 8, or p43 subunit of caspase 8. As a positive control, NF-κB elements p65/p50 were used as indicated. Results are representative of three independent experiments. (C) Jurkat T cells were transfected with HIV LTR or HIV LTR missing the KB motifs (HIV LTR Δ KB), along with TK-Renilla as a transfection control, and the Casp8p43 with or without IKKγ, dominant negative (DN) IκBα as indicated. Data are expressed as Luciferase units, normalized to TK-Renilla and representative of three independent experiments. (D) 293T cells were transfected with the indicated constructs. The following morning nuclear extracts were prepared and incubated with a P32 labeled HIV LTR NF-κB probe in the presence or absence of antibodies to NF-κB proteins p50 and p65. Then complexes were run on a 6% nondenaturing gel, and analyzed using autoradiography.

Recently, caspase 8 and certain of its homologs (caspase 10, c-FLIP, FADD) have been identified as modulators of the NF-κB response [21]. Because caspases are critical to apoptosis induced by HIV, we questioned whether they or their cleavage products also might initiate HIV transcription. First, we confirmed that gp120 treatment results in procaspase 8 cleavage (Figure 3A). Consequently, we generated plasmids expressing full length caspase 8, or the Casp8p43 cleavage product, and assessed their ability to cause transactivation of a HIV LTR luciferase reporter construct. In such experiments, we observed that the Casp8p43 cleavage product was a more potent inducer of HIV LTR activity than full length caspase 8 (Figure 3B). Moreover, this effect is inhibited by coexpression of the regulatory subunit (IKKγ) of the IKK signaling complex or a super repressor form of IKBα (Figure 3C). Casp8p43 failed to transactivate the HIV LTR with deletions of the NF-κB binding domains (ΔκB), which indicates that Casp8p43-mediated transactivation of the wild-type HIV LTR is mediated specifically by NF-κB. Finally, EMSA analysis confirmed Casp8p43 driven NF-κB activation (Figure 3D) induced by the p50/p65 heterodimer, as indicated by inhibition by p50 or p65 antibodies. Such data link apoptosis signaling to NF-κB activation, in a manner that requires caspase cleavage (as Casp8p43 is only present after caspase 8 activation), potentially as a homeostatic attempt of a dying cell to block its own death by initiating NF-κB driven activation of antiapoptotic regulatory proteins. Moreover, our hypothesis provides and explanation for prior observations [22]: Jurkat T cells stably transfected with HIV LTR-Luc were stimulated to die by ultraviolet (UV) irradiation and LTR driven luciferase expression measured. HIV transcription was induced by UV, and also was inhibited by a pan-caspase inhibitor (Z-VAD-Fmk), indicating that HIV LTR activation by UV requires caspase activation [22]. Because UV irradiation causes apoptosis by activating a caspase 8 dependent death pathway [23], these data are consistent with our hypothesis that caspase 8 cleavage intermediates drive HIV replication. In contrast, apoptosis induced by staurosporin (a direct mitochondriotoxin, which does not activate caspase 8), induced high levels of death, but did not activate the HIV LTR [23].

### HIV Infection of Caspase 8 Deficient T Cells Results in Reduced Apoptosis and Impaired HIV Replication

**Figure 4:**
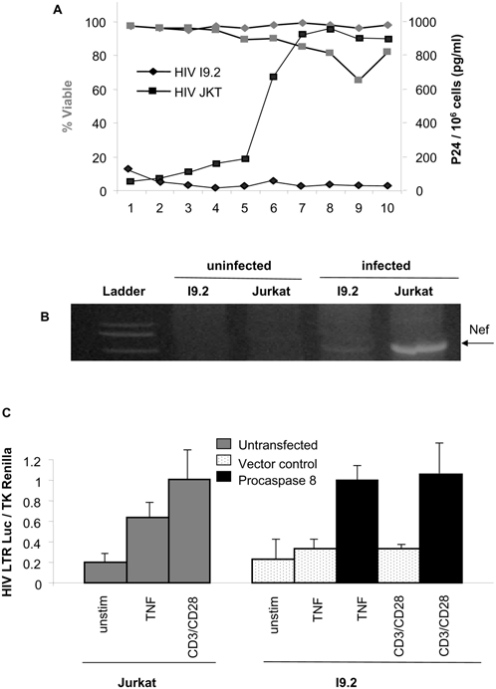
Optimum HIV replication requires caspase 8. (A) Jurkat T cells (squares) or I9.2 cells (diamonds) were infected with HIV IIIb, and analyzed daily for viability (red) and for P24 production (blue). (B) Two days following infection, cells were harvested, and DNA extracted. The DNA was assayed for HIV Nef by PCR. (C) Non-transfected Jurkat T cells or I9.2 cells transfected with control vector or procaspase 8, and stimulated with TNF or CD3/CD28 as indicated were analyzed for HIV LTR driven expression normalized to Renilla.

Given our experimental data suggesting that the caspase 8 cleavage product p43 drives HIV transcription, we opted to assess the relevance of this observation during experimental HIV infection. Since caspase inhibitors do not prevent procaspase 8 autoprocessing which generates p43 [24], we used I9.2 cell lines deficient in caspase 8. We confirmed expression of both CD4 and CXCR4 in I9.2 cells (data not shown). Consistent with previous observations, infection of Jurkat T cells results in significant apoptosis that peaks on day 9, and high levels of viral replication, as measured by HIV p24 production. By contrast, and as would be predicted if caspase 8 were essential for both HIV-induced apoptosis and HIV replication, infection of I9.2 cells resulted in less death and less viral replication (Figure 4A). Successful infection of these cells was confirmed by DNA PCR for HIV Nef, indicating that the initial events of attachment, insertion and reverse transcription occur in both Jurkat and I9.2 cells (Figure 4B), albeit to a lesser degree in the I9.2 cells. We therefore directly assessed whether caspase 8 is required for HIV replication by using a system that does not depend on HIV infection. Jurkat T cells or caspase 8-deficient I9.2 cells were transfected with a luciferase reporter construct under control of the HIV LTR, along with TK-Renilla as a control for transfection efficacy. These cells were then stimulated with agents known to induce HIV replication: TNF or CD3/CD28 crosslinking and luciferase measured. Whereas, Jurkat T cells responded to such treatment with robust luciferase expression, I9.2 cells did not produce significant luciferase in response to TNF or CD3/CD28. However, when I9.2 cells were first reconstituted with procaspase 8, I9.2 cells became responsive to both TNF and CD3/CD28 stimulation of the HIV LTR (Figure 4C). These data demonstrate that caspase 8 can restore HIV LTR-driven transcription in caspase 8 deficient I9.2 cells.

## Discussion

Our observations that caspase 8 is required for optimum NF-κB dependent gp120 mediated HIV LTR activation, and that the Casp8p43 cleavage product is a more potent activator of HIV LTR than full length caspase 8 offers insights into the biology of apoptosis. That Casp8p43 more efficiently drives HIV LTR activity than does full length caspase 8 is implicitly intuitive since all cells contain full length caspase 8, and it would be potentially harmful for all cells to have NF-κB activation. However, upon receipt of a death stimulus, NF-κB activation likely represent a compensatory reaction of that dying cell in an attempt to upregulate KB dependent antiapoptotic gene transcription. In an HIV-infected cell, the compensatory NF-κB response has the additional effect of stimulating HIV LTR transcription, and HIV replication. Furthermore, it is tempting to speculate that this death initiated NF-κB response is a reason why HIV evolved to be regulated transcriptionally by NF-κB.

When applied to HIV pathogenesis, such a model of apoptosis resulting in NF-κB activation allows the prediction that inhibiting caspase 8, and consequently inhibiting apoptosis, might result in two independent beneficial outcomes for HIV-infected patients. First, since causes of CD4 T cell loss include exaggerated rates of apoptosis, induced by a variety of viral and host stimuli, inhibiting caspase 8 activation would likely reduce the rate of CD4 T cell loss. In addition, and perhaps unexpectedly, our current data suggest that caspase 8 inhibition might have the additional benefit of reducing viral replication. Attempts to therapeutically modify apoptosis *in vivo* have principally focused on promoting apoptosis for cancer therapy. Our observations suggest an additional reason to explore inhibition of apoptosis in cells from HIV-infected patients.

We have recently described a novel pathway of apoptosis initiated by HIV, wherein HIV Pr, which is present and active within the cystosolic fraction, interacts and cleaves procaspase 8, creating a novel, HIV specific caspase 8 fragment, we call Casp8p41 [25]. Casp8p41 has two independent functions: induction of mitochondrial dependent apoptosis [21], [26], and activation of NF-κB [26]. Therefore, both gp120 and HIV protease each contribute to enhanced HIV replication indirectly through NF-κB activation, both in a caspase 8 dependent manner (albeit different mechanisms of caspase 8 activation). It will be of interest to determine whether other HIV specific proteins (eg., Tat and Nef) which have also been ascribed the two seemingly independent activities of apoptosis induction and enhanced viral replication, might also be linked via caspase 8.

Knowledge that gp120 independently drives viral replication offers insights into the means by which HIV achieves viral burdens which sometimes exceed 10^6^ /ml: new viral particles initiate a positive feedback loop, by acting upon infected cells to activate NF-κB, enhance HIV transcription and produce even more progeny viruses. In situations where this positive feedback loop is unopposed (e.g., in the absence of antiviral immunity or antiviral therapy), it becomes clear why viral burdens are highest immediately post infection, and then reduce slightly to individuals own ‘viral setpoint’. This positive feedback loop likely also occurs in settings of drug interruptions; it takes several days to make the first round of new virus, thereafter the activating effects of gp120 (and likely other proteins) ensure that the slope of viral rebound is steep. Ultimately the viral setpoint in that setting reflects the opposing forces of apoptosis driven HIV replication versus antiviral immunity and any impairment in replicative fitness caused by drug resistance. These data therefore have clear implications for the therapeutic strategies aimed at reactivating HIV from latency; consideration must be given to agents (perhaps gp120) which activate procaspase 8. Finally, data in the current report suggest that extreme caution be used for proposed therapeutic vaccine strategies which plan to use env, as enhanced replication might promote disease progression and drug resistance.
